# The Role of Histone Modifications in the Pathogenesis of Diabetic Kidney Disease

**DOI:** 10.3390/ijms24066007

**Published:** 2023-03-22

**Authors:** Christodoula Kourtidou, Konstantinos Tziomalos

**Affiliations:** First Propedeutic Department of Internal Medicine, AHEPA Hospital, Medical School, Aristotle University of Thessaloniki, 54636 Thessaloniki, Greece

**Keywords:** diabetes mellitus, chronic kidney disease, diabetic kidney disease, histone modification, oxidative stress, inflammation, fibrosis

## Abstract

Diabetic kidney disease (DKD) is the leading cause of chronic kidney disease. The pathogenesis of DKD is multifactorial, with several molecular pathways implicated. Recent data suggest that histone modification plays an important role in the development and progression of DKD. Histone modification appears to induce oxidative stress, inflammation and fibrosis in the diabetic kidney. In the present review, we summarize the current knowledge on the association between histone modification and DKD.

## 1. Introduction

Chronic kidney disease (CKD) affects 9.1% of the population worldwide and is an important cause of morbidity and mortality [1]. Diabetic nephropathy is the leading cause of CKD and end-stage renal disease (ESRD) [2]. Even in prediabetes, there is evidence of kidney dysfunction [3]. The decline of estimated glomerular filtration rate (eGFR) and increased urinary albumin excretion are accompanied with an increased risk for progression to ESRD and death [4]. Moreover, CKD is a risk factor for cardiovascular disease (CVD) [5].

Epigenetic mechanisms include DNA methylation, histone modification, and microRNA (miRNA) [6]. Histone modification has been involved in the pathogenesis of diabetes mellitus but emerging data suggest that it also plays a role in the pathogenesis of DKD [7,8]. In the current review, we summarize the current evidence regarding the association between histone modifications and DKD. Of note, histone modification appears to be implicated in other microvascular complications of diabetes mellitus, i.e., neuropathy and retinopathy, and also in the pathogenesis of atherosclerosis in these patients [8], but these associations are outside of the scope of the present review.

## 2. Pathogenesis of DKD

The defining histopathologic features of DKD are glomerular basement membrane (GBM) thickening, mesangial matrix expansion, nodular glomerulosclerosis and tubular and vascular lesions [9]. p21 plays a key role in glomerular and mesangial cell hypertrophy [10]. The pathophysiology of DKD is characterized by impaired renal hemodynamics, oxidative stress, inflammation and a dysregulated renin–angiotensin–aldosterone system (RAAS) (Table 1 and Table 2) [11]. Glomerular hyperfiltration in diabetes predisposes to albuminuria, a decline in renal function and progressive kidney damage [12,13]. In addition to hyperglycemia, glomerular hyperfiltration in diabetic patients is also attributed to hyperaminoacidemia, hypertension and obesity [13]. Hyperinsulinemia stimulates endothelin 1 (ET-1) secretion in the endothelium and in turn increased ET-1 levels induce vasoconstriction which eventually leads to vascular dysfunction [14].

Intrarenal oxidative stress is another feature of DKD [15,35]. Oxidative stress predisposes to inflammation and renal fibrosis in DKD [15]. In diabetes, thioredoxin interacting protein (Txnip) is involved in the pathogenesis of oxidative stress, podocyte injury and apoptosis [16,17]. Mitochondrial injury in diabetes is present in renal tubular cells, podocytes, mesangial cells, and glomerular endothelial cells [36]. In turn, glomerular endothelial mitochondrial dysfunction might increase the risk for DKD [37]. Moreover, in the podocytes of DKD patients, mitochondrial dynamics are impaired [38].

Autophagy represents a mechanism of preservation of podocyte integrity [39]. Compromised autophagy is implicated in the pathogenesis of DKD [22,23]. Autophagy interacts with advanced glycation end-products (AGEs), oxidative stress, endoplasmic reticulum (ER) stress, RAAS activation and renal fibrosis in DKD [22,23]. mTORC1 negatively regulates autophagy by inhibiting the activity of the unc-51-like autophagy activating kinase 1 (ULK1) complex through direct phosphorylation [22]. Insulin also impairs autophagic activity in proximal tubule epithelial cells [24].

DKD is also characterized by the accumulation of extracellular matrix (ECM) proteins, collagen, laminin and fibronectin, in the glomerulus and the renal tubulointerstitium, which ultimately lead to glomerulosclerosis and tubulointerstitial fibrosis [40]. The pathogenesis of diabetic renal fibrosis involves several players, including transforming growth factor β (TGF-β), mitogen-activated protein kinase (MAPK), Wnt/β-catenin, phosphatidyl-inositol-3 kinase (PI3K)/Akt, JAK/STAT and Notch pathways, which also interact with each other [25]. Plasminogen activator inhibitor-1 (PAI-1) promotes ECM accumulation by regulating fibrinolysis and plasmin-mediated matrix metalloproteinase activation and is strongly induced in various forms of kidney diseases including DKD [26]. In patients with type 2 diabetes mellitus, PAI-1 levels were inversely linked to renal function [41].

Several immune cells of both the innate and adaptive immune system also contribute to renal injury in DKD [18,19]. In addition, cellular adhesion molecules including intracellular adhesion molecule 1 (ICAM-1), vascular cell adhesion molecule 1 (VCAM-1) and E-selectin, chemokines including monocyte chemoattractant protein-1 (MCP-1), and cytokines including interleukin-6 (IL-6) are inflammatory mediators of renal injury in DKD [19]. MCP-1 levels have been shown to predict renal disease progression in diabetic patients [42]. Nuclear factor kappa-light-chain-enhancer of activated B cells (NF-κB) is a transcriptional factor that regulates the expression of inflammatory cytokines, chemokines and cellular adhesion proteins that are involved in DKD [20]. The nucleotide binding and oligomerization domain-like receptor family pyrin domain-containing 3 (NLRP3) inflammasome, by interacting with the mitogen-activated protein kinase (MAPK), induces oxidative stress, NF-κB signaling pathways and inflammatory factors, thus resulting in impaired kidney functioning and structural changes that culminate in DKD development and progression [21].

Advanced glycation end-products (AGEs) induced by hyperglycemia play an important role in the pathogenesis of DKD [43,44]. AGEs appear to promote renal inflammation and fibrosis in DKD [44].

In patients with diabetes mellitus, the production of TGF-β is increased in the kidney, which then facilitates the development of early manifestations of DKD [45]. TGF-β was shown to be upregulated in the glomerular and tubulointerstitial compartments of diabetic animal models [46]. Moreover, TGF-β is involved in the pathogenesis of renal hypertrophy, glomerulosclerosis and tubulointerstitial fibrosis observed in DKD [47]. The TGF-β/Smad signaling plays a crucial role in renal fibrosis, inflammation [48,49] and podocyte apoptosis [50].

Epithelial-to-mesenchymal transition (EMT), which occurs in tubular cells after injury, refers to a change in phenotype that is characterized by the conversion of epithelial markers to mesenchymal features and generates kidney fibrosis [51]. The downregulation of e-cadherin is observed in EMT [52]. It has been reported that endothelial-to-mesenchymal transition (EndMT) in glomeruli, a subtype of EMT, might be involved in the pathogenesis of DKD [53,54].

## 3. Histone Modification and DKD

Histone modifications regulate gene transcription, DNA repair and DNA replication [55,56]. Post-translational modifications (PTMs) occur at the residues of N-terminal tails of the histone proteins while modifications are also detected in the core regions of the histones [57]. The major histone PTMs are acetylation, methylation, ubiquitination and phosphorylation [58].

There is accumulating evidence that PTMs are involved in the regulation of genes associated with the pathogenesis of diabetes [7,8]. The PTMs of several genes related to diabetes and inflammation have been identified in monocytes and lymphocytes under diabetic conditions [59,60]. The persistent expression of genes and phenotypes created by former exposure to hyperglycemia, despite the subsequent achievement of glycemic control, a phenomenon termed metabolic memory, might be caused by epigenetic mechanisms and be associated with DKD [61,62]. Several studies demonstrated the non-histone modifications of histone-modifying enzymes in the setting of DKD [63,64,65].

## 4. Histone Acetylation and DKD (Table 3)

Histone acetylation can unravel chromatin and enable the binding of transcription factors and cofactors that lead to gene expression [55]. Histone acetylation, such as H3K9Ac, H3K14Ac and H4KAc, is generally correlated with permissive gene expression, while histone deacetylation is mainly associated with gene transcriptional repression [66]. Histone acetylation is catalyzed by histone acetyltransferases (HATs) and histone deacetylation by histone deacetylases (HDACs) [55]. p300/CREB-binding protein (CBP) and p300/CBP-associated factor (PCAF) are some of the known HATs [67]. Among the four classes of HDACs, the sirtuins (Sirt 1–7) belong in the third class [67].

Studies in db/db diabetic mice reported that early glomerulosclerosis was associated with reduced histone H3K9 and H3K23 acetylation [68]. Other studies showed that acetylation levels of H3K9, H3K18 and H3K23 were increased in the kidneys of diabetic mice [29,69]. Moreover, H3K27ac levels in diabetic mice are increased compared with non-diabetic mice [70]. Moreover, the increased H3K9/14Ac levels in the diabetic kidney mainly involved the nuclei of both glomerular and renal tubular cells [71]. Others indicated that hyperglycemia induced histone acetylation at the H3K18 and H3K9/14 sites in mesangial cells [72].

**Table 3:** Effects of histone acetylation on the pathogenesis of diabetic kidney disease.

Histone Acetylation	Gene/Molecule Target	Implicated Mechanism	Histone Acetyltranserases/Deacetylases or Their Inhibitors
H3K9/14Ac	Promoters of PAI-1 and p21 genes in mesangial cells	ECM accumulation and hypertrophy	p300/CBP [31]
	Fibrinonectin protein expression in mesangial cells	Renal fibrosis	Lys-CoA [72]
	BMP-7 gene promoter in tubular cells	Renal fibrosis	SFN [27]
H3K18Ac	MCP-1, ICAM-1, VCAM-1 and iNOS in mesangial cells	Mesangial cell inflammation	Apelin [29]
H3K9Ac	MCP-1, ICAM-1, VCAM-1 and iNOS in mesangial cells	Mesangial cell inflammation	Apelin [29]
	Promoters of Notch1 and Notch4 in hyperglycemia-treated podocytes	Apoptosis and inflammation of podocytes	Sirt6 [33]
	Timp1 promoter in proximal tubular cells miR-29a proximal promoter	Tubular fibrosis	Sirt6 [73]
		Podocyte apoptosis	HDAC4 [34]

PAI-1: Plasminogen activator inhibitor-1, ECM: extracellular matrix, p300/CBP: p300/CREB-binding protein, BMP-7: bone morphologic protein 7, SFN: sulforaphane, MCP-1: monocyte chemoattractant protein-1, ICAM-1: intracellular adhesion molecule 1, VCAM-1: vascular cell adhesion molecule 1, iNOS: inducible nitric oxide synthases, Sirt6: sirtuin 6.

## 5. Histone Acetylation and Renal Inflammation in DKD

In the context of hyperglycemia, increased H3K9 and H3K18 acetylation levels in the mesangial cells were associated with the production of inflammatory mediators including MCP-1, ICAM-1, VCAM-1 and inducible nitric oxide synthases (iNOS) [29]. Furthermore, ChIP assays revealed that PAI-induced enrichment of H3K18ac at the promoters of ICAM-1 and MCP-1 was mitigated after PCAF knockdown along with altered transcription levels [69]. In vitro studies demonstrated elevated levels of H3K9ac in the promoters of Notch1 and Notch4 in hyperglycemia-treated podocytes, whereas Sirt6 suppressed Notch1 and Notch4 transcription by deacetylating histone H3K9 and protected podocytes from apoptosis and inflammation through inhibition of the Notch pathway [33].

## 6. Histone Acetylation and Renal Fibrosis in DKD

The enrichment of H3K9/14Ac at the promoters of PAI-1 and p21 genes in rat mesangial cells exposed to hyperglycemia and glomeruli from diabetic mice were associated with the increased expression of these genes [31]. In addition, the elevated H3K9/14Ac levels on the gene promoters of connective tissue growth factor (CTGF), PAI-1 and fibronectin 1 (FN-1) in the kidneys of diabetic mice were associated with activation of HAT p300/CBP [71]. ChIP assays found elevated levels of acetylated H3 at the promoter sites of the ET-1, FN genes in human umbilical vein endothelial cells (HUVECs) [74]. Under hyperglycemic and hyperinsulinemic conditions in kidneys the, levels of histone H3 acetylation in the fibrillin 1 gene were increased [75]. In another study in renal tubular cells, exposure to hyperglycemia augmented the enrichment of H3K18Ac, H3K27Ac and H3K4Me3 but not H3K9Ac on the collagen gene promoters [76]. Claudin-1 expression was regulated through the deacetylation of histone H3 and H4 by Sirt1 while Claudin-1 expression in podocytes induced podocyte effacement and resulted in albuminuria [77]. Sirt6 increased H3K9 acetylation in the promoter region of the pro-fibrotic factor Timp1 leading to the upregulation of Timp1 that may inactivate matrix metalloproteinases (MMPs) and reduced fibrous tissue degradation [73]. In another study, 12(*S*)-hydroxyeicosatetraenoic acid (12(*S*)-HETE), an oxidized lipid which promotes oxidative stress, induced profibrotic gene expression in association with the augmented enrichment of their promoter by H3K9Ac and H3K4me in rat mesangial cell [78]. In mesangial cells, the inhibition of H3K9/14 acetylation by Lys-CoA, an inhibitor of CBP/p300 HAT activity, led to diminished FN protein expression [72]. In vitro, trichostatin A (TSA)-induced acetylation of H3 was correlated with the overexpression of E-cadherin and decreased expression of the FN gene in TGF-β1-treated tubular epithelial cells [79]. In addition, TSA treatment increased the acetylation of histones H3 and H4 in the E-cadherin promoter, H3 in the Id2 promoter and H4 in the bone morphologic protein 7 (BMP-7) promoter and subsequently regulated the expression of Id2 and BMP-7 [80]. In another study, sulforaphane (SFN) treatment counteracted the diabetes-induced decline in H3K9/14Ac expression and H3K9/14Ac levels in the BMP-7 gene promoter, which was accompanied by BMP-7 upregulation [27].

## 7. Histone Acetylation and Apoptosis in DKD

Blockade of HDAC4 signaling increased the H3K9Ac levels in the miR-29a proximal promoter and miR-29a transcription in hyperglycemia-treated podocytes and attenuated the hyperglycemia-induced apoptosis [34]. In another study, the acetylation of histone H4 in the glucose-regulated protein (GRP78) promoter, was increased in the DKD group, compared with controls; valproate reversed apoptosis by increasing the acetylation of histone H4 in the GRP78 promoter and decreasing the acetylation of histone H4 in the C/EBP-homologous protein (CHOP) promoter, two promoters that encode proteins related to ER stress [81].

## 8. Histone Acetylation and Other Manifestations of DKD

Hyperglycemia-induced Txnip expression was associated with the enrichment of H3K9ac at the promoter region of the gene in the kidneys of diabetic mice [82]. ChIP assays showed increased levels of H3K18Ac and H2BK5Ac in the membrane metalloendopeptidase (Mme) promoter region in the kidneys of diabetic rats but enrichment of H2AK5Ac remained unchanged on Mme promoter in kidneys [83]. The expression of ULK1 in diabetic mice was decreased compared with that in non-diabetic mice, whereas ChIP assays also revealed reduced levels of H3K27Ac on of the ULK1 promoter sequences in diabetic mice [84].

## 9. Histone Methylation and DKD (Table 4)

Histone methylation takes place in arginines, lysines and histidines on histone tails [85]. Histone methylation modifications promote or repress gene transcription [86]. Lysine methylation is catalyzed by methyltransferases (KMTs) and is reversed by demethylases (KDMs) [87,88,89]. SET domain-containing enzymes are one class of KMTs [87].

KDM6A expression was increased and H3K27me2/3 levels were decreased in the kidneys of diabetic mice [90] and in hyperglycemia-treated podocytes and renal tubular cells [91,92]. In addition, in kidney tissues from patients with DKD, there was a reduction of podocyte, glomerular cell and tubular cell H3K27me3 [93]. Elevated H3K4me1 and H3K4me3 levels and decreases in H3K27me3 levels were detected in the kidneys of diabetic animals compared with non-diabetic controls [94].

**Table 4:** Effects of histone methylation on the pathogenesis of diabetic kidney disease.

Histone Methylation	Gene/Molecule Target	Implicated Mechanism	Histone Methyltranserases/Demethylases or Their Inhibitors
H3K9me2/3	p21 gene promoter in rat mesangial cells	Mesangial cell hypertrophy	SET7/9 [32]
H3K4me1/2/3	p21 gene promoter in rat mesangial cells	Mesangial cell hypertrophy	SET7/9 [32]
H3K4me3	Txnip promoter in mesangial cells	Renal inflammation	C646 [95]
	Spp1 gene hyperglycemia-treated mesangial cells		MM-102 [94]
H3K4me1	MCP-1 promoter in db/db mice	Renal inflammation	SET7/9 [30]
	Txnip promoter in mesangial cells	Renal inflammation	C646 [95]
H3K27me3	Promoters of IL1b and IL6 in renal mesangial cells	Renal inflammation	Palmitic acid, KDM6A [90]
	FN and PAI-1 expression	Mesangial cell hypertrophy	KMT6A [96]
	HES1 promoter hyperglycemia-treated mesangial cells	Mesangial cell proliferation and fibrosis	KMT6A [97]
	α-SMA, collagen I, and FN in rat renal interstitial fibroblast cells	Renal fibrosis	KMT6A [28]
	Pax6, TxnIP promoters in renal podocytes	Podocyte oxidative stress	KMT6A [98]

MCP-1: monocyte chemoattractant protein-1, Spp1: secreted phosphoprotein 1, IL1b: interleukin 1b, IL6: interleukin 6, KDM6A: lysine demethylase 6A, FN: fibronectin, PAI-1: plasminogen activator inhibitor-1, KMT6A: histone-lysine N-methyltrasferase, HES1: enhancer of split 1, α-SMA: smooth muscle alpha-actin, Pax6: paired box 6.

## 10. Histone Methylation and Renal Inflammation in DKD

Palmitic acid (PA) stimulation decreased the enrichment of H3K27me3 on the promoters of IL1b and IL6, whereas the overexpression of KDM6A further decreased H3K27me3 levels on their promoters in renal mesangial cells [90]. In contrast, under PA treatment, KDM6A knockdown increased the enrichment of H3K27me3 on the promoters of IL1b and IL6 [90]. In vitro studies in renal tubular cells exposed to hyperglycemia suggest that the secretion of IL6 and MCP-1 increases and inflammation stimulated the expression of KMT1A which catalyzed H3K9me3 to inhibit the transcription of inflammatory genes [99]. In another study, SET domain-containing protein 8 (SETD8) overexpression reduced hyperglycemia-induced IL-1β and IL-18 expression by attenuating hyperglycemia-induced MARK4 expression and inactivating the NLRP3 inflammasome, whereas H4K20me1, a downstream target of SETD8, was enriched in the MARK4 promoter region in HUVECs [100]. Furthermore, ChIP assays demonstrated that H4K20me1 levels were increased at the promoter region of E26 transformation-specific sequence transcription factor-1 (ESE-1), in HUVECs exposed to hyperglycemia [101]. Set7-induced enrichment of H3K4m1 on NF-kB p65 gene promoter led to the upregulation of ICAM-1, MCP-1, iNOS and cyclooxygenase-2 (COX-2) in peripheral blood monocytes [102]. Others reported that SET7/9 recruitment and H3K4me1 levels at MCP-1 promoters in diabetic mice were markedly higher compared with non-diabetic animals; the ER stress resulted in the enrichment of H3K4me1 at MCP-1 promoter in the kidneys of diabetic db/db mice through the preferential induction of SET7/9 [30]. Finally, renal hypertrophy in diabetes was linked to the upregulation of profibrotic and proinflammatory genes and reduced levels of H3K27me3 and KMT6A in these gene promoters [103].

## 11. Histone Methylation and Renal Fibrosis in DKD

The levels of H4K20me1, a downstream target of KMT5A, decreased in hyperglycemia-treated HUVECs and the levels of H4K20me1 were enriched in the promoter region of enolase 1 (ENO1), regulating the ENO1 levels and thereby inducing EndMT [104]. On the other hand, the expression of e-cadherin was decreased and its promoter methylation was upregulated with increasing KDM6A expression, but there was no relationship with the level of H3K27me2/3 in the E-cadherin promoter [92]. Hyperglycemia decreased KMT1A and histone H3K9me3 levels in the FN and p21 gene promoters [105]. On the other hand, the augmented expression of KMT1A increased the H3K9me3 levels in the FN and p21 promoters and protected against hyperglycemia-induced cell hypertrophy [105]. Hyperglycemia increased levels of KMT6A, resulting in the enrichment of H3K27me3 at the deptor promoter leading to decreased deptor expression, which led to the activation of mTORC1 and mTORC2, controlled mesangial cell hypertrophy and FN and PAI-1 expression [96]. In accordance with these findings, KMT6A inhibited mesangial cell proliferation and fibrosis-related protein expression in hyperglycemia-treated mesangial cells by downregulating hairy and enhancer of split 1 (HES1) expression; a ChiP assay showed that the KMT6A and H3K27me3 levels were increased in the HES1 promoter and enrichment was intensified by KMT6A overexpression [97]. Moreover, EZH2 knockdown significantly decreased the levels of H3K27me3 and downregulated the levels of smooth muscle alpha-actin (α-SMA), collagen I, and FN in rat renal interstitial fibroblast cells, whereas the silencing of KMT6A repressed the TGFβ1-stimulated expressions of α-SMA, collagen I, and FN [28]. In animal models of type 1 diabetes mellitus, the levels of H3K27m3 were reduced in MCP-1, vimentin and the fibrosis marker Fsp1 genes, while the levels of H3K4m2 were increased [106]. More recently, it was reported that histone H3 K79 methyltransferase Dot1l has an antifibrotic effect by repressing *Edn1*, which encodes endothelin-1 [95]. Moreover, Dot1l interacts with HDAC2 to regulate *Edn1* transcription [95]. Notably, HDAC2 was also shown to protect against renal ischemia/reperfusion injury by suppressing *Edn1* transcription [98].

## 12. Histone Methylation and Oxidative Stress in DKD

In the kidneys of diabetic mice, hyperglycemia-induced Txnip expression was associated with the stimulation of activating histone marks H3K4me3 and H3K4me1, as well as with a decrease in the repressive histone mark H3K27me3 at the promoter of the gene [95]. In another study, KMT6A downregulated TxnIP expression through H3K27me3 enrichment in the promoter region of the transcription factor paired box 6 (Pax6), whereas the depletion of KMT6A led to an increase in the hyperglycemia-induced reactive oxygen species (ROS) levels and cellular death [98]. In addition, the depletion or inhibition of KMT6A attenuated the increase of ROS in hyperglycemia-treated human renal tubular epithelial cells, while the KMT6A knockdown attenuated the reduced expression of silent information regulator 1 (SIRT1) by hyperglycemia treatment [107]. The restoration of SIRT1 expression was in line with the elimination of H3K27me3 from the SIRT1 promoter and the inhibition of intracellular ROS levels [107].

## 13. Histone Methylation and Glomerular and Mesangial Hypertrophy in DKD

TGF-β1 stimulated the expression of the p21 gene in rat mesangial cells, while the levels of H3K9me2/3 and H3K4me1/2/3 at p21 gene promoter were correlated with p21 gene expression [32]. Similarly, the reduced H3K9me2 level in the p21 gene promoter in the glomeruli of type 1 diabetic rats was inversely correlated with p21 gene upregulation [108]. The increased H3K4me3 level in secreted phosphoprotein 1 (Spp1), the osteopontin gene, promoter were associated with Spp1 gene enhanced expression in hyperglycemia-treated human mesangial cells, whereas the levels of histone marks were linked to increases of Spp1 expression in animal studies [94]. Diminished podocyte H3K27me3 was correlated with glomerular injury while H3K27me3 controlled Notch signaling and podocyte dedifferentiation [93].

## 14. Histone Methylation and Other Manifestations of DKD

SIRT-6 induction of activated protein kinase (AMPK) signaling, through H3K9 and H3K56, contributed to hyperglycemia-induced mitochondrial dysfunction and apoptosis in podocytes [109]. In another study, ChIP assays demonstrated the enrichment of H3K4me1 levels at the MAP4K3 gene and the upregulation of MAP4K3 in diabetic mice [110]. MAP4K3 knockdown decreased HYPERGLYCEMIA-induced apoptosis while MAP4K3 downregulation counteracted the expression of apoptosis markers [110]. Histone H2AK119 and H2BK120 ubiquitination regulated H3K4Me2 and H3K9Me2 chromatin marks by modifying the expression of their respective HMTs, SET7/9 and KMT1A [111]. The increased expression of SET7/9 and decreased expression of KMT1A could be associated with the increased enrichment of H3K4Me2, and decreased H3K9Me2 level at Collagen Type I Alpha 1 Chain (Col1a1) gene promoter and increased Col1a1 gene expression in the diabetic kidneys [111].

## 15. Conclusions

Histone modification appears to play an important role in the pathogenesis of DKD. Indeed, histone acetylation and methylation are implicated in the propagation of inflammation, oxidative stress and fibrosis in the kidney. Even though the exact mechanism(s) through which impaired epigenetic landscape contributes to DKD is still unclear, it appears that an altered enhancer landscape plays an important role [112,113,114]. It remains to be established whether targeting this pathway might prevent the development or delay the progression of DKD. Of note, it is well established that DKD is associated with an increased risk for CVD [115]. In this context, somatic mutations, particularly in DNA methyltransferase 3 alpha and TET methylcytosine dioxygenase 2, are also implicated in the pathogenesis of both atherosclerosis and DKD, partly by a pro-inflammatory effect [116,117].

## Figures and Tables

**Table 1:** Major risk factors for kidney disease in patients with diabetes mellitus and their management.

Risk Factor	Target	Recommended Management
Hyperglycemia	HbA_1c_ < 7% (<6.5% if achievable without a substantially increased risk for hypoglycemia, or 7.5–8.0% in patients with substantial comorbidities or reduced life expectancy)	Sodium glucose transporter inhibitors Other antidiabetic agents if needed
Hypertension	Systolic blood pressure < 140 mmHg (<120 mmHg if it can be reasonably achieved) Diastolic blood pressure 80–90 mmHg	Angiotensin converting enzyme inhibitors or angiotensin receptor blockers Finerenone Other antihypertensive agents if needed
Obesity	At least 5% weight loss from baseline	Physical activity and low-calorie diet (500 kcal/day deficit)
Dyslipidemia	Low density lipoprotein cholesterol levels < 55 mg/dl and LDL-C reduction > 50% from baseline	Statins Ezetimibe and proprotein convertase subtilisin/kexin type 9 inhibitors if needed

**Table 2:** Principal mechanisms implicated in the pathogenesis of diabetic kidney disease.

Mechanism	Associated Mechanisms	Regulators
Impaired renal hemodynamics	Hyperglycemia, hyperaminoacidemia, hypertension and obesity [13]	ET-1 [14]
Oxidative stress	Inflammation and renal fibrosis [15]	Txnip [16,17]
Dysregulation of the immune system/ Inflammation	Immune cells and adhesion molecules [18,19]	NF-κB [20], NLRP3 [21]
Compromised autophagy	AGEs, oxidative stress, ER stress, RAAS activation and renal fibrosis [22,23]	mTORC1 [22], insulin [24]
Fibrosis	Oxidative stress [15], autophagy [22,23]	TGF-β, MAPK, Wnt/β-catenin, PI3K/Akt, JAK/STAT, and Notch pathways [25], PAI-1 [26]
Histone modification	Fibrosis [27,28], inflammation [29,30], hypertrophy [31,32], apoptosis [33,34]	

ET-1: endothelin 1, Txnip: thioredoxin interacting protein, NF-κB: nuclear factor kappa-light-chain-enhancer of activated B cells, NLRP3: nucleotide binding and oligomerization domain-like receptor family pyrin domain-containing 3, AGEs: advanced glycation end-products, ER: endoplasmic reticulum, RAAS: renin–angiotensin–aldosterone system, TGF-β: transforming growth factor β, MAPK: mitogen-activated protein kinase, PI3K: phosphatidyl-inositol-3 kinase, PAI-1: plasminogen activator inhibitor-1.

## Data Availability

No new data were created in this paper.
